# Effects of dietary nisin supplementation on the growth performance, serum biochemistry, digestive enzyme activities, intestinal morphology, and intestinal microbiota in rabbits

**DOI:** 10.3389/fvets.2025.1726365

**Published:** 2025-12-11

**Authors:** Xinyu Wu, Tiancheng Shao, Yee Huang, Xuemei Cui, Yanfang Luo, Quanan Ji, Zizhe Hu, Sheng Teng, Guolian Bao, Yan Liu

**Affiliations:** 1 Institute of Animal Husbandry and Veterinary Science, Zhejiang Academy of Agricultural Sciences, Hangzhou, Zhejiang, China; 2 Key Laboratory of Microbiological Metrology, Measurement & Bio-Product Quality Security, State Administration for Market Regulation, College of Life Sciences, China Jiliang University, Hangzhou, Zhejiang, China; 3 State Key Laboratory for Quality and Safety of Agro-Products, Institute of Animal Husbandry and Veterinary Sciences, Zhejiang Academy of Agricultural Sciences, Hangzhou, China

**Keywords:** nisin, rabbits, growth performance, intestinal health, antibiotic alternative

## Abstract

**Introduction:**

This study evaluated the impact of dietary supplementation with varying doses of nisin (NI) on the growth performance, serum biochemical parameters, intestinal digestive enzyme activity, short-chain fatty acid (SCFA) profiles, mucosal morphology, and the cecal microbiota composition in rabbits.

**Methods:**

Healthy female New Zealand white rabbits (5 weeks old; *n* = 90) of comparable body weight were randomly allocated to five groups: a positive control (PC) group receiving a basal diet supplemented with kitasamycin (300 mg/kg), three NI groups supplemented with nisin at 600 (NI600), 800 (NI800), or 1,000 (NI1000) mg/kg, and a negative control (NC) group receiving the basal diet without additives. Each treatment was comprised of three replicates (*n* = 6 per replicate), and the trial lasted 42 days.

**Results:**

The results showed that the rabbits administered NI displayed significantly enhanced final body weights as compared to the NC group (*P* < 0.05), with a dose-dependent effect. Notably, the NI800 and NI1000 groups exhibited a superior average daily gain (ADG) and average daily feed intake (ADFI). Serum analyses showed improved lipid profiles and elevated antioxidant enzyme activities concomitant with reduced lipid peroxidation in the NI-supplemented groups. Enzymatic assays indicated elevated duodenal a-amylase activity in the NI800 group as compared to the PC (*P* < 0.05) and enhanced ileal trypsin activity in the NI800 as compared to NI1000 and PC (*P* < 0.05). Histological evaluation confirmed that the NI800 group displayed optimal intestinal villi morphology, characterized by increased density, height, and structural integrity relative to the PC and NC controls. Metagenomic analysis of the cecal microbiota further revealed dose-dependent shifts in the diversity and composition of the microbiota, with the NI800 group exhibiting pronounced restructuring. Enriched functional pathways in the NI groups, including cofactor/vitamin metabolism, amino acid biosynthesis, energy homeostasis, and environmental adaptation.

**Discussion:**

Collectively, these findings highlight that NI supplementation enhances digestive efficiency, augments systemic antioxidant defenses, fortifies intestinal barrier function, and modulates microbial ecology and SCFA production, there by promoting growth and metabolic health in rabbits. Nisin, especially at 800 mg/kg, demonstrates significant potential as an antibiotic alternative.

## 1 Introduction

Rabbit meat possesses exceptional nutritional value. As non-ruminant herbivores with a simple monogastric system, rabbits are capable of digesting a wide variety of natural grasses and leaves (1, 2). Dietary fiber constitutes a major component of their diet, with roughage accounting for nearly 50% of the complete feed formulation consumed by rabbits (3). Fiber plays a crucial role in stimulating gastrointestinal peristalsis, influencing the rate of chyme passage, and supporting the production of volatile fatty acids (VFAs) through microbial fermentation in the caecum, thereby helping to maintain intestinal health (4). According to previous studies, digestive tract disorders are among the most prevalent health issues in rabbit production (5, 6). Weaned rabbits, in particular, are susceptible to digestive disturbances due to their underdeveloped intestinal walls. These disturbances often manifest as diarrhea or bloating, leading to impaired growth performance (7) and imposing substantial economic losses on rabbit production systems. Recent studies have prioritized feed additives capable of enhancing intestinal health and growth efficiency, aligning with sustainable animal several agriculture goals, such as ensuring food safety, minimizing antibiotic reliance, and mitigating environmental degradation (8). While antibiotic utilization in livestock has historically enhanced productivity and disease control (9, 10), the indiscriminate use of antibiotics has been reported to cause antimicrobial resistance, immune dysfunction, and ecological harm, in addition to residual antibiotic contamination in animal products (11–14). Consequently, global regulatory frameworks restrict antibiotic feed supplements (15), spurring demand for novel, non-toxic, residue-free alternatives (16).

Nisin (NI), a well-characterized bacteriocin, exhibits potent inhibitory effects against pathogenic bacteria in food systems (17–19). As postbiotics, bacteriocins are ribosomally synthesized, post-translationally modified peptides, enabling direct genomic identification (20). Importantly, bacteriocins, such as NI, selectively target pathogens while preserving commensal gut microbiota (21), positioning them as promising antibiotic substitutes in both human therapeutics and veterinary medicine (22). The United States Food and Drug Administration classifies NI as “Generally Recognized as Safe” (GRAS; 21 CFR 184.1538), highlighting its regulatory acceptance (23). Structurally, NI is a 34-amino acid polycyclic antimicrobial peptide active mainly against gram-positive bacteria. Initially utilized as a food preservative (approved by the FDA and European Union), NI has emerged as a viable antibiotic alternative in agriculture (23–25).

Evidence indicates that NI enhances poultry growth performance by regulating the ecology of the gut microbiota, attenuating inflammation, and reducing enterocyte apoptosis (23). Józefiak et al. (26) observed dose-dependent effects of NI (100, 300, 900, and 2,700 IU/g) in broilers, although there are few *in vivo* studies assessing its impact on avian productivity (27). Consequently, the European Food Safety Authority (EFSA) has advocated for the further evaluation of NI as a therapeutic agent for livestock (28). This study was aimed at elucidating the influence of NI on the following factors in growing rabbits: (1) growth kinetics; (2) serum biochemical profiles and antioxidant capacities; (3) intestinal digestive enzyme activities; (4) intestinal mucosal morphology and tight junction gene expression; and (5) cecal microbiome composition and function.

## 2 Materials and methods

### 2.1 Experimental animals and housing conditions

Female New Zealand white rabbits (Oryctolagus cuniculus; *n* = 90; 5 weeks old; mean body weight = 1.2 ± 0.1 kg) were purchased from Tongfeng Rabbit Farm (Fuyang District, Hangzhou, China). Animals were acclimated in a climate-controlled facility (temperature: 20–30 °C; humidity: 60–70%) under a 12-h light/dark cycle for 6 weeks in. Rabbits were housed in triplicate cohorts (*n* = 6 per replicate) within a three-tiered cage system (7 m × 5 m), with *ad libitum* access to feed and water. Cages were sterilized with 0.1% sodium hypochlorite prior to experimentation. All procedures complied with the ethical guidelines of Zhejiang Agricultural Ethics Association and the Farm Animal Governance Committee of Zhejiang Province (Approval Number: 1935).

### 2.2 Experimental design and diets

Rabbits were stratified by body weight and randomized to five dietary regimens (*n* = 18 per group; 3 replicates of 6 rabbits): (1) Negative Control (NC) received only the basal diet (Table 1); (2) NI600 received the basal diet + 600 mg/kg NI (6 × 10^5^ U/g; Shandong Youyide Biotechnology Co., Ltd., China); (3) NI800 received the basal diet + 800 mg/kg NI; (4) NI1000 received the basal diet + 1000 mg/kg NI; and (5) Positive Control (PC) received the basal diet + 300 mg/kg kitasamycin (antibiotic control).

**Table 1:** Composition and nutrient levels of the basal diet (air-dried basis).

**Diet composition**	**Percentage**	**Nutrient levels**	**Content**
Corn	28.00	Digestible energy (MJ/kg)	10.28
Wheat bran	14.50	Crude protein (%)	15.70
Bean meal (43% crude protein)	7.00	Methionine and cystine (%)	0.49
Alfalfa meal	34.70	Calcium (%)	1.03
Malt root	10.00	Total phosphorus (%)	0.56
Soybean oil	2.50	Lysine (%)	0.72
Limestone	0.80	Crude fiber (%)	13.11
NaCl	0.50		
Premix	2.00		

Per kilogram of premix: 100 mg of iron, 10 mg of copper, 30 mg of manganese, 60 mg of zinc, 0.2 mg of iodine, 0.15 mg of selenium, 0.1 mg of cobalt, 6,000 IU of vitamin A, 1,000 IU of vitamin D, 50 mg of vitamin E, 5 mg of pantothenic acid, folic acid 20 mg, and choline 50 mg.

NI doses (600, 800, and 1,000 mg/kg) were selected based on prior studies in poultry and our own preliminary trials in rabbits, which indicated that this range effectively modulates gut microbiota without adverse effects. The doses were designed to bracket the anticipated effective level to reliably determine the dose-response relationship and identify the optimal dosage. The 42-day trial included weekly monitoring of feed intake (FI) and body weight (BW). Growth metrics, such as average daily gain (ADG), average daily feed intake (ADFI), and feed conversion ratio (FCR), were calculated. ADFI, ADG, and feed-to-gain (F/G) ratio were calculated as follows: ADFI = total feed intake/experimental days; ADG = (FBW – IBW)/experimental days; and F/G = ADFI/ADG.

### 2.3 Blood and tissue harvesting

On days 21 and 42, rabbits from each experimental group (*n* = 6/group) were fasted for 12 h, anesthetized via auricular vein injection (pentobarbital sodium, 30 mg/kg), and euthanized by exsanguination. Whole blood was collected from the carotid artery and centrifuged at 3,000 × g for 10 min at 4 °C. The serum was collected and stored at −80 °C. Segments (5 cm) of duodenum, jejunum, and ileum were excised, rinsed in ice-cold PBS, flash-frozen in liquid nitrogen, and preserved at −80 °C for downstream assays.

### 2.4 Serum biochemical and immunological assays

Serum was analyzed for urea nitrogen (UN), total cholesterol (TC), albumin (ALB), globulin (GLO), and total protein (TP) using a GS200 automated analyzer (Genius Electronics, China). The antioxidant capacity of the serum was measured using glutathione peroxidase (GSH-Px), superoxide dismutase (SOD), and malondialdehyde (MDA) ELISA kits (Jiangsu EnzymeFree Industrial Co., Ltd., China). Levels of IL-6, TNF-α, and IFN-γ were quantified using specific ELISA kits (Lianke Biotech Co., Ltd., Hangzhou, China) according to the manufacturer's instructions, and the absorbance was measured using a SpectraMax M5 microplate reader (Molecular Devices, Shanghai, China).

### 2.5 Intestinal morphometry and digestive enzyme activities

Tissue samples from the duodenum, jejunum, and ileum were fixed in 4% paraformaldehyde for 24 h. Following fixation, the samples were trimmed, dehydrated, cleared, and embedded in paraffin. Subsequently, the embedded tissues were sectioned, stained, and mounted onto glass slides.For morphological assessment, sections were examined under a light microscope at 400 × magnification. Fields with intact tissue morphology and clear orientation were selected for analysis. The villus height (VH) and crypt depth (CD) were measured using the Image-Pro Plus software (version 6.0; Media Cybernetics, Inc., Maryland, USA). For each intestinal section, at least five intact villi and their corresponding crypts were randomly selected for measurement. The mean values of V and C were calculated, and the villus height to crypt depth (V/C) ratio was determined. Mucosal scrapings were homogenized in PBS (Solarbio, Beijing, China), centrifuged (2,500 × g, 10 min), and the supernatants were assayed for α-amylase, trypsin, and chymotrypsin activity using ELISA kits Lianke Biotech Co., Ltd., Hangzhou, China).

### 2.6 Short-chain fatty acid (SCFA) analysis

To ensure representative sampling, the entire cecal content from each rabbit was thoroughly homogenized. Then, a sample (500 mg) were weighed into a sterile 1.5 mL microcentrifuge tube, homogenized with 1 mL of ultrapure water, and centrifuged at 1,400 × g for 10 min at 4 °C). The supernatant was mixed with crotonic acid solution, snap-frozen, and stored at −20 °C for at least 24 h. After thawing on ice, the samples were filtered through a 0.22-μm membrane (NEST, Wuxi, China), and the SCFA levels were quantified using a Shimadzu GC-2010 Plus a gas chromatograph (GC-2010 Plus, Shimadzu, Kyoto, Japan).

### 2.7 Tight junction gene expression analysis

Total RNA was extracted from intestinal tissues using TRIzol reagent (GENEray, Shanghai, China), and both the quality and concentration were assessed. Complementary DNA (cDNA) was synthesized using a SweScript RT SuperMix kit (Servicebio, Wuhan, China). Quantitative real-time PCR (qPCR) was conducting utilizing SYBR Green Master Mix on a LightCycler^®^ 480 system (Roche Diagnostics, Basel, Switzerland). Primer sequences for target genes (ZO-1, occludin, claudin-1) and the reference gene (GAPDH) are listed in Table 2 (Sangon Biotech, Shanghai, China). Cycling parameters were: 1 cycle at 95 °C for 30 s, followed by 40 cycles of 94 °C for 15 s and 60 °C for 30 s. Relative mRNA expression was calculated using the 2^−Δ*ΔCt*^ method normalized to GAPDH. Detect the gene primer sequence as presented in Table 2.

**Table 2:** Primer sequences used for quantitative real-time PCR analysis.

**Gene**	**Prime sequence (5'-3')**	**Length (bp)**	**Accession**
*ZO*-1-F	CCTGCGAAACCCACCAAA	293	XM_008269782.1
*ZO*-1-R	ATGCTGTCGAAAGGTCAGGG		
*Claudin-F*	GCAAGAGGCCGTATCCAGAG	193	NM_001089316.1
*Claudin-R*	AGTCCGTCTCGTAGTGGTCT		
*Occludin-F*	AGTGATTCGGATTCTGTCTATGC	112	XM_008262320.1
*Occludin-R*	ACTTCCCAGTAAGCCAGTTCC		
*GAPDH-F*	TGTTTGTGATGGGCGTGAA	129	NC_013676.1
*GAPDH-R*	CCTCCACAATGCCGAAGT		

### 2.8 Cecal microbiota profiling

Microbial DNA was extracted from cecal contents using Hipure Soil DNA Kit (Magen, Guangzhou, China). The V3-V4 hypervariable region of the 16S rRNA was amplified using primers 341F and 806R (Table 3). PCR conditions were: 1 cycle of 95 °C for 5 min, followed by 30 cycles of 95 °C for 1 min, 60 °C for 1 min, 72 °C for 1 min, and a final extension at 72 °C for 7 min. Amplicons were purified using AMPure XP Beads (Beckman Coulter Life Sciences, Indiana, USA), quantified utilizing a Qubit 3.0 Fluorometer (Thermo Fisher Scientific, Massachusetts, USA), and paired-end sequenced (2 × 250 bp) on an Illumina NovaSeq 6000 platform (Illumina, California, USA). Operational taxonomic units (OTUs; 97% similarity) were annotated against the SILVA database (v138) using Mothur software package (version 1.48.0).

**Table 3:** 16S PCR primers used in this study.

**Type**	**Region**	**Primer**	**Sequence (5^′^-3^′^)**	**Length**
16S	V3-V4	341F	CCTACGGGNGGCWGCAG	466
		806R	GGACTACHVGGGTATCTAAT	

### 2.9 Statistical analysis

All data are presented as the mean ± standard error of the mean (SEM). Statistical comparisons between groups were performed by one-way analysis of variance (ANOVA) followed by Fisher's Least Significant Difference (LSD) *post-hoc* test using SPSS software (version 26; IBM Corp., Armonk, NY, USA). A *p*-value of less than 0.05 was considered statistically significant (^*^p < 0.05, ^**^p < 0.01, ^***^p < 0.001). Graphs were generated using GraphPad Prism (version 9.4.1; GraphPad Software, San Diego, CA, USA).

## 3 Results

### 3.1 Growth performance

Dietary supplementation with varying doses of NI (NI600, NI800, NI1000) or antibiotics (PC) significantly enhanced the growth performance as compared to the NC group (Table 4). Initial body weights were comparable across all groups (*p* > 0.05). Final body weights displayed a dose-dependent increase in the NI groups (*p* < 0.05). The ADG was significantly elevated in the NI800 and NI1000 groups as compared to the NC group (*p* < 0.05). The ADFI was significantly increased in the NI600, NI800, and NI1000 groups compared to NC and PC groups (*p* < 0.05). The F/G trended toward a decrease with increasing NI doses, but differences did not reach statistical significance (*p* > 0.05).

**Table 4:** Effects of dietary treatments on the growth performance of rabbits.

**Parameter**	**NC**	**NI600**	**NI800**	**NI1000**	**PC**	***P*-value**
IW (g)	1086.6 ± 48.9	1110.5 ± 54.5	1068.3 ± 108.0	1063.8 ± 43.5	1082.5 ± 87.5	0.829
FW (g)	2155.4 ± 208.8^b^	2351.5 ± 109.4^a^	2452.2 ± 139.9^a^	2500.8 ± 60.16^a^	2397.3 ± 219.9^a^	0.015
ADG (g)	25.4 ± 5.0^c^	29.5 ± 2.8^bc^	32.9 ± 3.4^ab^	34.2 ± 1.8^a^	31.3 ± 4.7^ab^	0.007
ADFI (g)	112.1 ± 5.2^c^	127.0 ± 2.5^b^	134.3 ± 3.1^a^	136.1 ± 1.2^a^	123.4 ± 6.7^b^	0.000
F/G	4.51 ± 0.69	4.32 ± 0.33	4.11 ± 0.36	3.99 ± 0.19	4.01 ± 0.61	0.301

Rabbits in the negative control (NC) group were fed a basal diet without any additives. Rabbits in the NI600 group were fed the basic diet supplemented with 600 mg/kg nisin (NI). Rabbits in the NI800 group were fed the basic diet supplemented with 800 mg/kg NI. Rabbits in the NI1000 group were fed the basic diet supplemented with 1,000 mg/kg NI. Rabbits in the positive control (PC) group were fed the basal diet supplemented with 300 mg/kg kitasmycin. Different superscript letters a, b and c indicate significant difference among all groups (*p* < 0.05). IW, initial weight; FW, final weight; ADG, average daily gain; ADFI, average daily feed intake; F/G, material to weight ratio; The data are expressed as the mean ± standard deviation (SE).

### 3.2 Comprehensive profiling of serum biochemical parameters in rabbits

**Figure 1:**
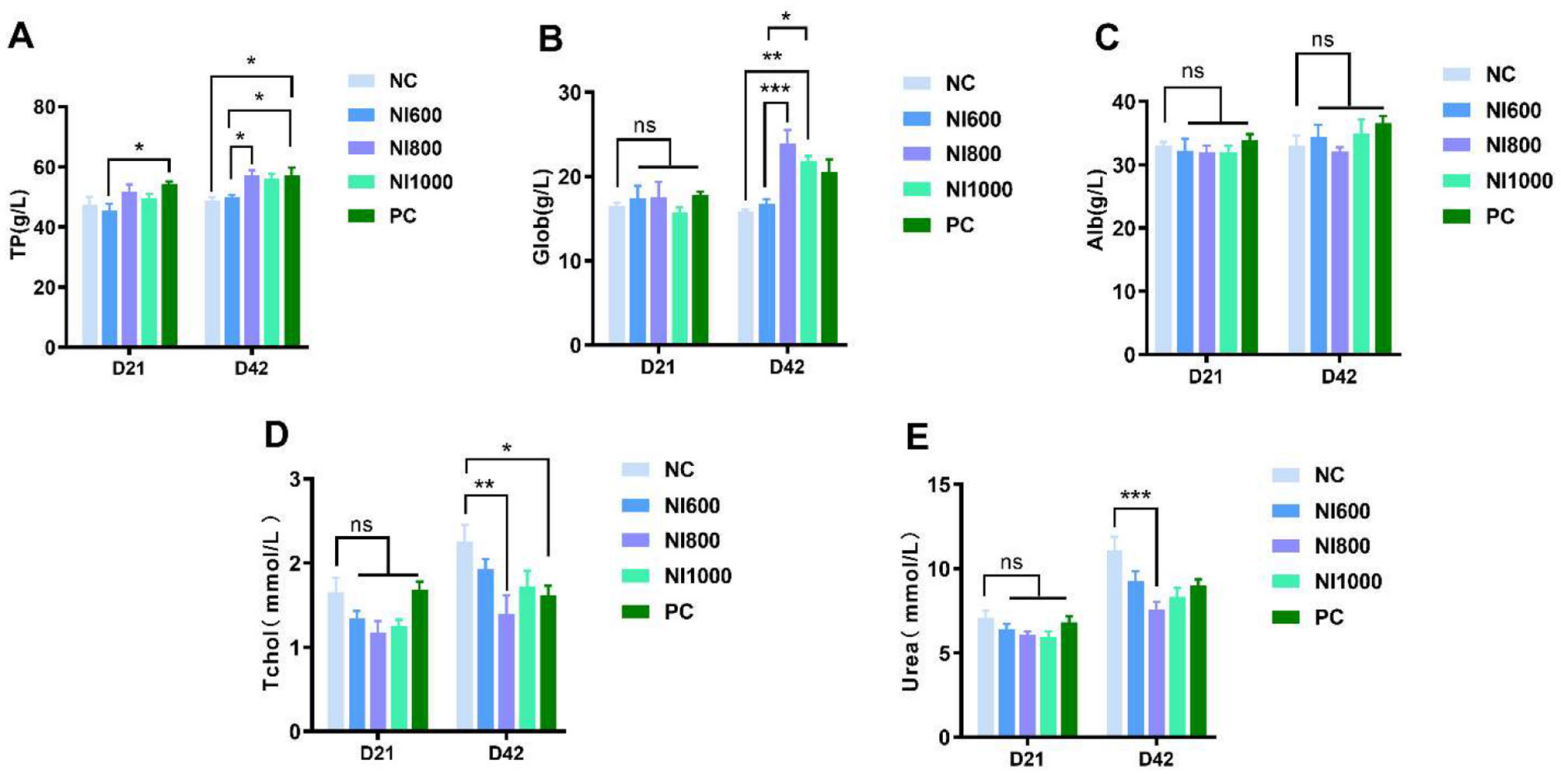
The effects of dietary nisin (NI) at 600 (NI600), 800 (NI800), and 1000 (NI1000) mg/kg and antibiotic (positive control; PC) supplementation on the serum biochemical parameters in rabbits. **(A–E)** The serum levels of TP (g/L), Glob (g/L), Alb (g/L), TChol (mmol/L), Urea (mmol/L). Data are expressed as the mean ± SE (*n* = 6). *p < 0.05, **p < 0.01, ***p < 0.001 indicate significant differences as compared to the negative control (NC) group; ns, not significant; TP, total protein; Glob, globulin; Alb, albumin; TChol, total cholesterol; Urea, urea nitrogen.

Serum biochemical parameters related to protein and lipid metabolism were assessed. As shown in Figure 1, no significant differences were observed in the TP or Alb concentrations on days 21 (D21) and 42 (D42) (*p* > 0.05), suggesting stable systemic protein synthesis and hepatic function across cohorts (Figures 1A, C). The NI600 group displayed a marked elevation in Glob levels as compared to the NC, NI800, and NI1000 groups (*p* < 0.01) until D42 (Figure 1B). The Tchol levels were substantially lower in all experimental groups as compared to the NC group on D21 (*p* < 0.05). This trend persisted on D42, with the NI800 and NI1000 groups exhibiting significantly lower Tchol than the NC group (*p* < 0.05) (Figure 1D). Urea concentrations on D42 were significantly reduced in all experimental groups and the PC group as compared to the NC group (*p* < 0.05) (Figure 1E).

### 3.3 Quantitative mapping of pro- and anti-inflammatory serum cytokines in rabbits

Quantification of serum proinflammatory cytokines identified dose-dependent immunomodulatory trends (Table 5). On D42, quantitative ELISA analysis found elevated, but not statistically significant, TNF-α concentrations across low-, medium-, and high-dose intervention groups as compared to NC and PC groups (*p* > 0.05). No significant differences were observed for IFN-γ or IL-6 levels (*p* > 0.05), suggesting selective cytokine modulation independent of Th1-type inflammatory responses.

**Table 5:** Effects of the various dietary interventions on the serum cytokines levels of rabbits.

**Parameter**	**NC**	**NI600**	**NI800**	**NI1000**	**PC**	***P*-value**
IL-6 (pg/mL)	136.42 ± 4.78	135.20 ± 6.72	138.03 ± 13.95	140.29 ± 5.13	145.29 ± 13.56	0.168
TNF-α (pg/ml)	327.42 ± 25.53	350.25 ± 21.54	368.31 ± 21.40	343.96 ± 38.59	328.47 ± 24.03	0.233
IFN-γ (μg/mL)	848.38 ± 81.25	861.42 ± 69.60	856.07 ± 53.96	857.76 ± 53.85	872.06 ± 73.59	0.991

IL-6, interleukin-6; TNF-α, tumor necrosis factor-α; IFN-γ, interferon- γ. Data are expressed as the mean ± standard deviation (SE).

### 3.4 Analysis of serum antioxidant capacity

Dietary supplementation with graded doses of NI (600, 800, and 1,000 mg/kg) elicited dose-dependent modulation of systemic antioxidant defenses in rabbits (Table 6). SOD activity was significantly higher in the NI800 group compared to the NC (*p* < 0.01) and PC (*p* < 0.05) groups, and it was significantly higher in the NI1000 compared to NC group (*p* < 0.05). GSH-Px levels were markedly upregulated in NI600 and NI1000 groups as compared to both the NC and PC groups (*p* < 0.05), indicating enhanced redox buffering capacity. MDA concentrations, an indicator of lipid peroxidation, was significantly reduced in all NI groups compared to the NC group (*p* < 0.01). Furthermore, the NI800 and NI1000 groups exhibited significantly lower MDA levels as compared to the NI600 group (*p* < 0.05), suggesting a non-linear dose-response relationship in mitigating oxidative damage.

**Table 6:** Effects of the various dietary treatments on the serum antioxidant indices of rabbits.

**Parameter**	**NC**	**NI600**	**NI800**	**NI1000**	**PC**	***P*-value**
SOD (U/mL)	15.48 ± 0.10^d^	15.49 ± 0.11^d^	19.7857 ± 0.14^a^	16.9909 ± 0.05^c^	19.41 ± 0.11^b^	0.000
GSH-Px (μmol/mL)	0.3888 ± 0.01^d^	0.7432 ± 0.01^a^	0.2958 ± 0.01^e^	0.7036 ± 0.01^b^	0.52 ± 0.01^c^	0.000
MDA (nmol/mL)	5.2387 ± 0.11^a^	3.4725 ± 0.09^b^	1.7740 ± 0.07^c^	1.7570 ± 0.08^c^	1.3148 ± 0.05^d^	0.000

a, b, c, d, and e mean values within a row with unlike superscript letters were significantly different (*p* < 0.05). The data are presented as the mean for the antioxidant index. GSH-Px, glutathione peroxidase; MDA, malondialdehyde; SOD, superoxide dismutase. The data are expressed as mean ± standard deviation (SE).

### 3.5 Comprehensive analysis of intestinal digestive enzyme activities

**Figure 2:**
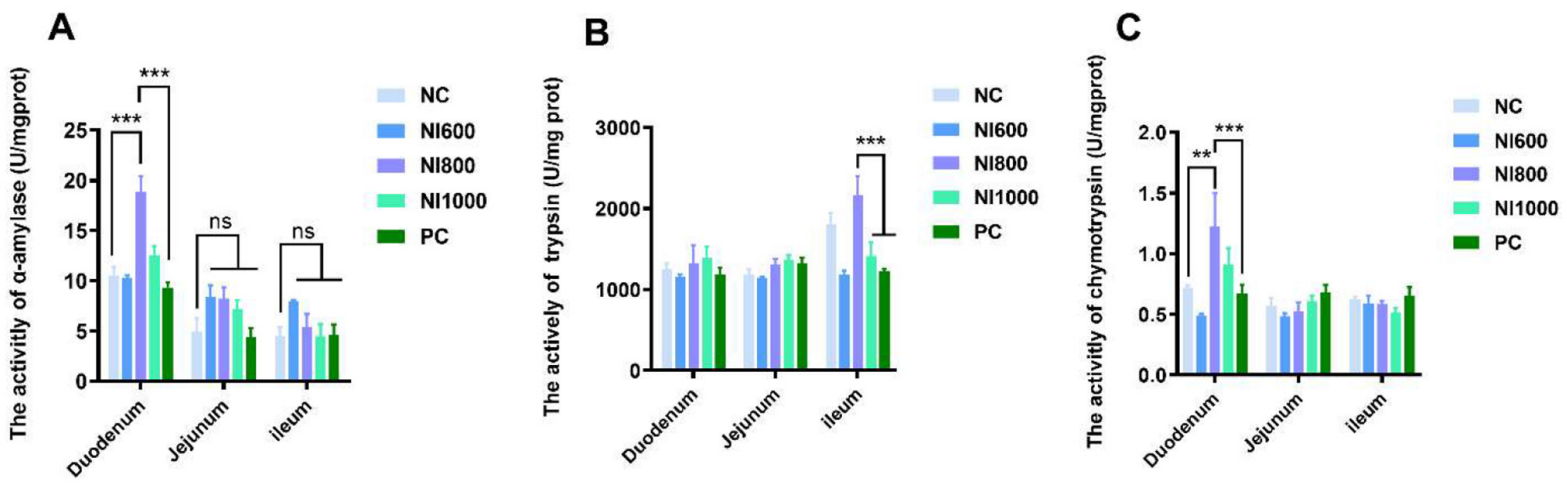
The effects of dietary nisin (NI) at 600 (NI600), 800 (NI800), and 1,000 (NI1000) mg/kg and antibiotic (positive control; PC) supplementation on digestive enzyme activities in the duodenum, jejunum, and ileum of rabbits. Enzyme activities are shown: **(A)** α-amylase, **(B)** trypsin, and **(C)** chymotrypsin. The data are expressed as the mean ± SEM (*n* = 6). *p < 0.05, **p < 0.01, and ***p < 0.001 indicate significant differences between groups within a segment.

The effects of dietary treatments on digestive enzyme activities in different intestinal segments are shown in Figure 2. In the duodenal, α-amylase activity and chymotrypsin activity in the NI800 cohort exhibited a significant elevation compared to the NC and PC groups (*p* < 0.05) (Figures 2A, C). In the ileum, trypsin activity was significantly elevated in the NI800 group as compared to all other groups (*p* < 0.05) (Figure 2B). The jejunum exhibited no significant alterations in enzymatic activities among different treatments (*p* > 0.05) (Figures 2A–C).

### 3.6 Revised morphological analysis of small intestinal mucosa in rabbits

**Figure 3:**
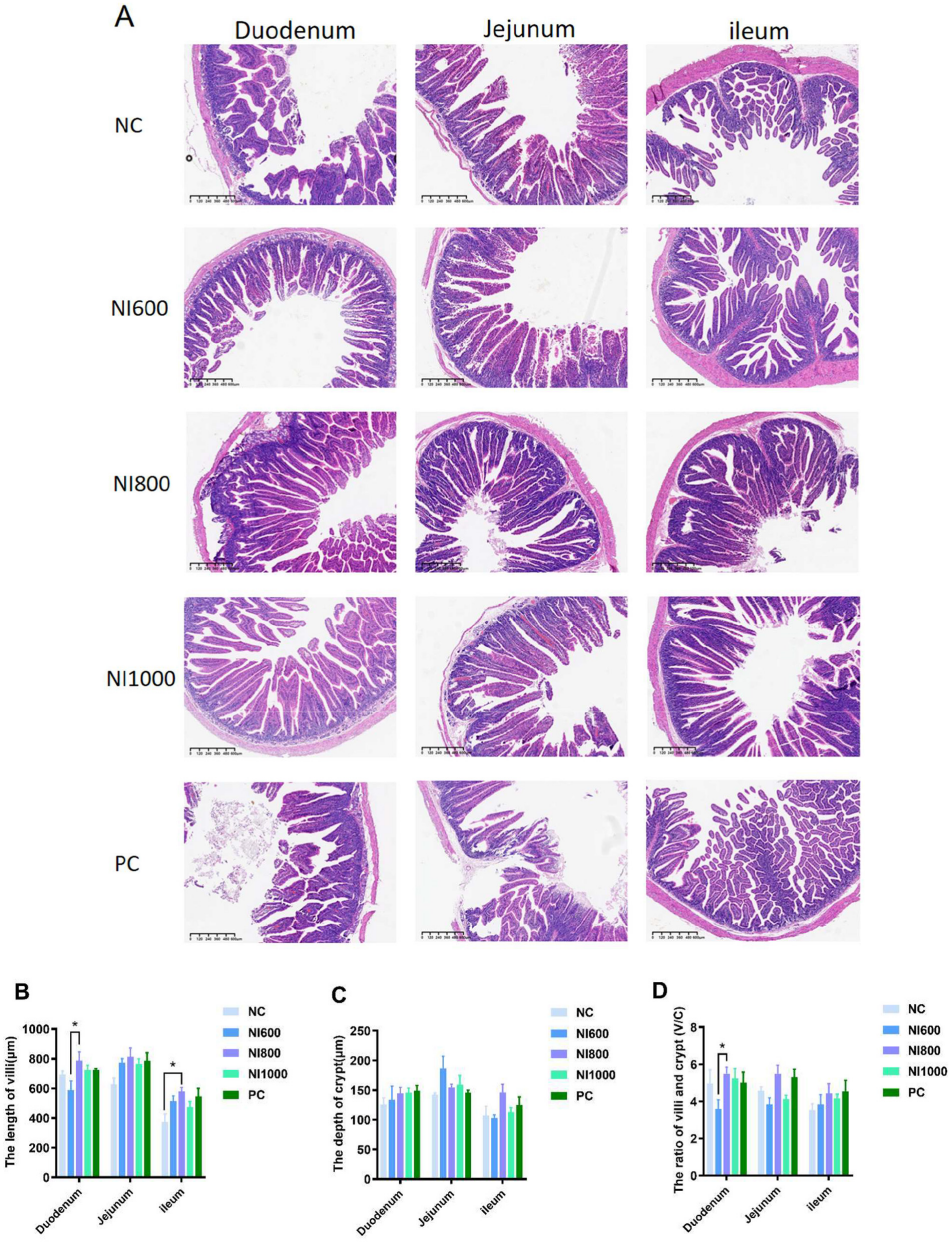
The effects of dietary supplementation with nisin (NI) or the positive control (PC) on the small intestinal mucosal morphology of rabbits. **(A)** Representative photomicrographs of H&E-stained histological sections from the duodenum, jejunum, and ileum (scale bar: 600 μm, 40 × magnification); **(B, D)** Morphometric analysis of the small intestinal mucosa: **(B)** Villus height (VH), **(C)** Crypt depth (CD), and **(D)** the VH-to-CD ratio in the duodenum, jejunum, and ileum. Data are presented as the mean ± SEM (*n* = 6). *p < 0.05, **p < 0.01, and ***p < 0.001 indicate significant differences between groups within a segment.

Representative photomicrographs of H&E-stained sections from each group are shown in Figure 3A, illustrating the general mucosal architecture upon which the morphometric analysis was performed. Quantitative assessment of the small intestine revealed statistically significant differences (*p* < 0.05) in villus height (VH), crypt depth (CD), and the VH/CD ratio among the experimental groups across the duodenum, jejunum, and ileum (Figures 3B–D). Consistent with these morphometric findings, qualitative histological examination further supported the beneficial effects of nisin supplementation. As evidenced in the representative micrographs, the jejunum and ileum from rabbits in the NI600, NI800, NI1000, and PC (antibiotic-treated) groups exhibited better-preserved villus structure, characterized by greater integrity and density, compared to the NC group. In contrast, the NC group and, to a lesser extent, the PC group displayed morphological indications of villus atrophy, including structural fragility and occasional epithelial disruption.

This study delineated the impact of dietary supplementation with NI600, NI800, NI1000, and PC on the cecal concentrations of SCFAs in rabbits, as quantified in Table 7. SCFAs, including acetic acid, propionic acid, isobutyric acid, butyric acid, isovaleric acid, and valeric acid, were assessed. Isobutyric acid, butyric acid, and isovaleric acid in the cecal contents of rabbits showed no statistically significant alterations among groups (*p* > 0.05). In contrast, the NI1000 group exhibited a significant elevation in acetic acid and valeric acid concentrations as compared to the NC, NI600, and NI800 groups (*p* < 0.05). The valeric acid concentration was significantly higher in the NI1000 and PC groups compared to the NC, NI600, and NI800 groups (*p* < 0.05). These findings collectively suggest that Nisin (NI) uniquely enhances the production of specific SCFAs, potentially through selective stimulation of saccharolytic or proteolytic bacterial taxa.

**Table 7:** The effects of dietary treatments on the cecal short-chain fatty acid concentrations (μmol/g).

**Parameter**	**NC**	**NI600**	**NI800**	**NI1000**	**PC**	***P*-value**
Acetic acid	3.85 ± 0.124^c^	4.14 ± 0.589^c^	5.43 ± 0.444^b^	7.17 ± 0.421^a^	8.41 ± 0.305	0.000
Propionic acid	0.50 ± 0.014^b^	0.51 ± 0.021^ab^	0.52 ± 0.062^ab^	0.56 ± 0.033^a^	0.73 ± 0.151	0.028
Isobutyric acid	0.05 ± 0.004^a^	0.05 ± 0.011^a^	0.05 ± 0.01^a^	0.06 ± 0.020^a^	0.07 ± 0.016	0.659
Butyric acid	0.46 ± 0.056^a^	0.49 ± 0.023^a^	0.50 ± 0.012^a^	0.50 ± 0.03^a^	0.51 ± 0.046	0.342
Isovaleric acid	0.20 ± 0.014^a^	0.21 ± 0.021^a^	0.21 ± 0.012^a^	0.25 ± 0.037^a^	0.22 ± 0.030	0.139
Valeric acid	0.03 ± 0.006^b^	0.04 ± 0.004^ab^	0.04 ± 0.004^ab^	0.06 ± 0.014^a^	0.06 ± 0.008	0.041

The data are presented as the mean ± SE. There was no significant difference between the non-alphabetic markers in the same line, and the difference between the superscript letters of a, b, c, and d indicated a significant difference (*p* < 0.05).

### 3.7 Analysis of tight junction protein mRNA expression in rabbits

**Figure 4:**
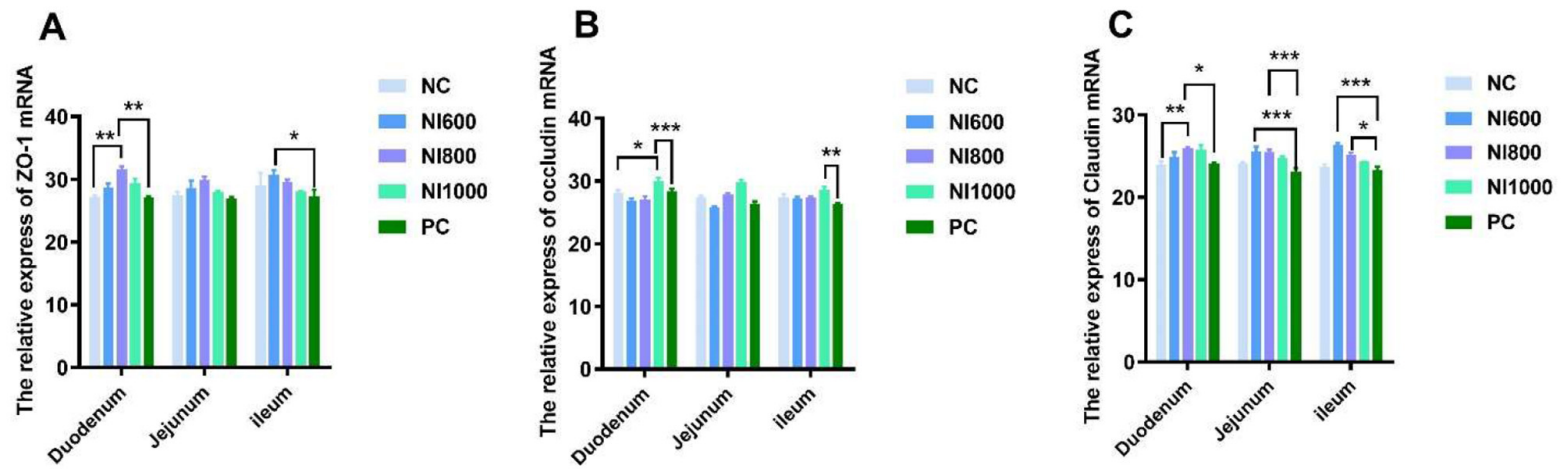
The effects of dietary nisin (NI) at 600 (NI600), 800 (NI800), and 1,000 (NI1000) mg/kg and positive control (PC) supplementation on the relative mRNA expression level of intestinal tight junction proteins. **(A)** Occludin, **(B)** Claudin-1, and **(C)** ZO-1 in the duodenum, jejunum, and ileum of rabbits. The data are expressed as the mean ± SEM (*n* = 6) relative to GAPDH using the 2^−Δ*ΔCt*^ method. *p* < 0.05 and *p* < 0.01 indicate significant differences between groups within a specific gene and segment. *p < 0.05, **p < 0.01, and ***p < 0.001 indicate significant differences between groups within a segment.

The relative gene expression of tight junction proteins, such as ZO-1, occludin, and claudin, in the three intestinal segments (duodenum, jejunum, and ileum) was assessed (Figure 4). A statistically significant difference (*p* < 0.05) was observed in the ZO-1 mRNA levels on the NI800 groups (Figure 4A). In the jejunum and ileum, the NI600 group displayed a marked upregulation of claudin mRNA as compared to the NC group (*p* < 0.05) and the PC group (*p* < 0.01) (Figure 4C). In the jejunum, the NI800 group showed a robust increase in claudin mRNA expression compared to the PC group (*p* < 0.01) (Figure 4C). The NI1000 group induced a striking upregulation of occludin mRNA in the jejunum as compared to other groups (*p* < 0.01) (Figure 4B).

### 3.8 Impact of NI on cecal microbial diversity and intestinal function

**Figure 5:**
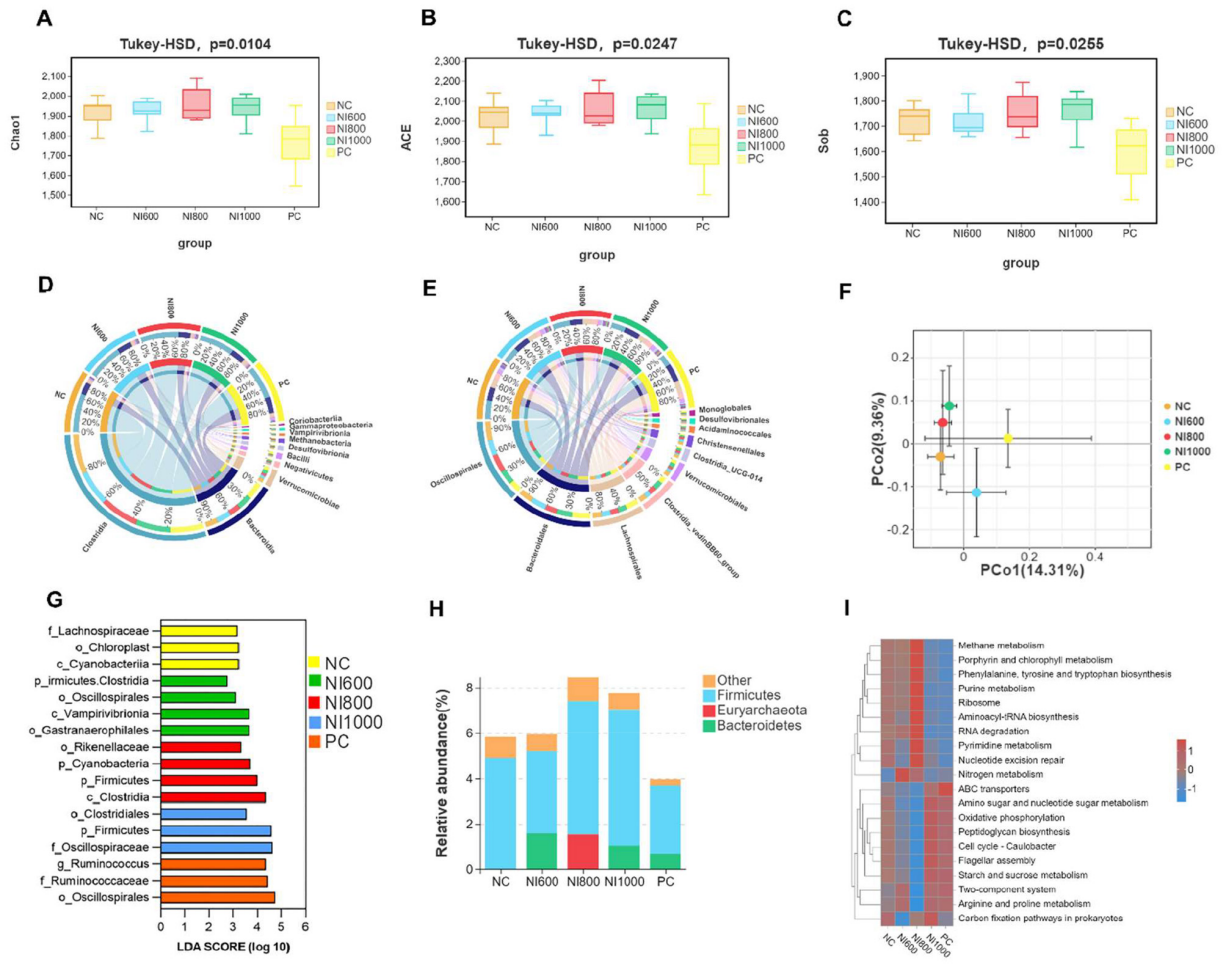
Impact of nisin (NI) on the cecal microbial diversity and intestinal function in rabbits. **(A–C)** Microbiota diversity analysis by Chao1, ACE, and Sob indices. **(D, E)** Analysis of the microbial composition at the phylum and genus levels. **(F)** Principal component analysis (PCA) revealed the distinct microbial community structures across sample types. **(G)** Linear discriminant analysis (LDA) evaluated the marker species. **(H)** Proportional representation of key species in distinct sample types. **(I)** Metabolic profiling of differentially abundant species via KEGG pathway analysis

NI supplementation significantly modulated the cecal microbial composition and intestinal function in rabbits (Figure 5). Alpha diversity indices (ACE, Chao1, and Sob) showed significant dose-dependent variations (*p* < 0.05), with the NI800 group exhibiting distinct clustering in beta diversity analysis (PCoA, Figures 5A–C) as compared to other groups. Taxonomic analysis revealed a predominance of Bacteroidetes and Clostridia at the class level (Figure 5D), while Spirochaetales, Bacteroidales, Lachnospira, and Clostridia_vadinBB60_Group dominated at the order level (Figure 5E). The beta diversity and LEfSe analyses further highlighted distinct phylogenetic demarcations (Figures 5F, G). Specifically, the negative control (NC) group was primarily characterized by a significant enrichment of taxa within the order Oscillospirales and the family Lachnospiraceae, including genera such as Ruminococcus and families Oscillospiraceae and Ruminococcaceae. These bacterial groups are widely recognized for their pivotal role in the degradation of complex dietary fibers and the subsequent production of short-chain fatty acids (SCFAs). In contrast, the cecal microbiota of the NI800 group exhibited a marked predominance of members belonging to the class Clostridia and the order Clostridiales under the phylum Firmicutes. As core constituents of the gut microbiome, clostridia are instrumental in protein fermentation and amino acid metabolism, and their enrichment in the NI800 group potentially reflects an adaptive microbial response to the heightened nutritional metabolic demands induced by niacinamide supplementation. Notably, the positive control (PC) group demonstrated a unique microbial profile, distinguished by a significant overrepresentation of the phylum Cyanobacteria, the class Cyanobacteriia, and sequences assigned to Chloroplast. The detection of these signatures is frequently attributed to the ingestion of plant-derived feed ingredients, suggesting that their pronounced abundance in the PC group may signify a distinct influence of undigested plant components from the basal diet on the gut microbiota composition. Functionally, Firmicutes emerged as a key contributor to intestinal metabolism, with its abundance scaling proportionally to NI dosage, suggesting a potential role in enhancing polysaccharide catabolism and SCFA production (Figure 5H). Metabolic profiling revealed upregulated energy, nucleic acid, and amino acid metabolism in the NI group, underscoring the capacity of NI to reprogram microbial metabolic networks and promote gut homeostasis (Figure 5I).

## 4 Discussion

Bacteriocins, which are secreted products of probiotics, are used in animal feed as a preventive against diseases and to promote animal health (29). As a polypeptide bacteriocin, NI is from *Lactococcus lactis subsp*. Related studies have demonstrated a positive impact of NI supplementation on the growth performance of broilers by increasing BW, feed intake, and simultaneously decreasing the FCR during the initial rearing period (days 1–14) (25). Additionally, a previous study (24) observed an improvement of 9.4% in the ADG of broiler rabbits fed with NI as compared to the control group. The present study comprehensively assessed the effects of dietary NI supplementation on the growth performance, serum biochemistry, intestinal health, and cecal microbiota composition in rabbits. The results revealed that NI, particularly at doses of NI800 and NI1000, significantly enhanced growth metrics, including the final body weight, ADG, and ADFI, as compared to the NC group. These findings align with previous studies in poultry, where NI supplementation improved growth performance by modulating the gut microbiota and enhancing nutrient absorption. Similar effects have been observed in other livestock species, suggesting a broader applicability of NI as a growth promoter. For example, research in piglets has shown that NI can improve growth performance and reduce the incidence of diarrhea (30).

Serum biochemical parameters can reflect the health status of animals (31). Under healthy conditions, the body maintains homeostasis through self-regulation. Measuring serum parameters helps assess the metabolic levels and nutrient utilization in rabbits, including Tchol, TP, Alb, Glob, and Urea levels. The results of serum biochemical parameters in this study demonstrated that, by the mid-experimental stage, Tchol values in all treatment groups were significantly lower than those in the NC group and also lower than the PC group. Notably, the NI800 and NI1000 groups displayed significantly reduced Tchol levels compared to the NC group on D42. Tchol, comprised of both HDL and LDL, serves as an indicator of lipid metabolism efficiency and liver health in rabbits. These results suggested that NI may positively influence lipid metabolism. Previous studies have demonstrated that certain bacteriocins can regulate cholesterol levels by interfering with cholesterol absorption in the intestine (32). Furthermore, previous research on bacteriocins and probiotic bacteria has suggested their potential to influence host lipid metabolism, potentially through mechanisms such as interfering with cholesterol absorption or modulating gene expression. No significant differences were observed in TP or Alb levels among groups on D21 and D42. However, on D42, the Glob value in the NI600 group was significantly higher than in the NC, NI800, and NI1000 groups. TP consists of Alb and Glob, reflecting protein metabolism in animals. Elevated TP levels suggest improved absorption of dietary protein. Alb, synthesized by the liver, is the most abundant plasma protein and maintains osmotic pressure, while Glob (primarily immunoglobulins) participates in immune functions (33–35). On D42, Urea levels in all treatment groups and the PC group were significantly lower than in the NC group. Urea, the end product of protein catabolism, is excreted via the kidneys. Elevated serum urea indicates excessive protein breakdown or impaired renal function, whereas reduced levels suggest efficient protein metabolism. These findings demonstrated that dietary supplementation with NI improved lipid profiles and protein metabolism in young rabbits.

Inflammation represents a cardinal biomarker of systemic health. Although proinflammatory cytokines (e.g., TNF-α, IFN-γ, IL-6) did not display statistically significant variations, the upward trend in TNF-α within the NI cohort suggests potential immunostimulatory effects via selective activation of host defense mechanisms. This cytokine-specific modulation may involve interactions of NI and innate immune pathways, particularly Toll-like receptor (TLR) signaling cascades (36). The absence of TH1-polarized inflammatory responses implies that the immunomodulatory activity of NI may be compartmentalized (e.g., within the gut-associated lymphoid tissue) rather than systemic. Concurrently, NI administration significantly upregulated the activity of antioxidant enzymes, including superoxide SOD and GSH-Px, while attenuating MDA levels, indicative of reduced oxidative stress. The NI800 group exhibited particularly robust SOD activity, surpassing both NC and PC groups. These observations align with the documented free radical-scavenging capacity of NI and its anti-lipoperoxidative properties (37). Similar antioxidant effects have been reported in other studies, where NI was shown to protect against oxidative damage *in vitro* (38). The dose-dependent suppression of MDA further corroborates the cytoprotective role of NI in maintaining membrane integrity.

Digestive enzymes are secreted by digestive glands and can catalyze various biochemical reactions, assisting the body in digesting and decomposing food ingested into the digestive tract. For instance, the digestion of starch and protein in food is primarily accomplished by α-amylase, trypsin, and chymotrypsin. The higher the activity of these enzymes, the higher the digestibility of nutrients, ultimately enhancing the growth performance of livestock and poultry (39, 40). The NI-dependent modulation of digestive enzymes displayed distinct regional specificity. For instance, NI800 significantly enhanced α-amylase activity in the duodenum, whereas NI600 elevated both α-amylase and trypsin activities in the ileum. This spatial variation likely reflects differential microbial colonization patterns and enzyme secretion dynamics along the gastrointestinal tract. Research has indicated that bacteriocins can influence the activity of digestive enzymes by altering the gut microbiota composition (41). The morphology of the small intestinal tissue can reflect the health status of the intestine and its digestive and absorptive functions. Aside from absorbing nutrients from food, the intestinal barrier also prevents harmful substances from entering the body. The height of the villi and the depth of the crypts, as well as their ratio, are usually regarded as key indicators (42, 43). The longer the villi, the more intestinal epithelial cells there are, and the stronger the ability to digest and absorb nutrients. The shallower the crypts, the slower the rate of cell generation at the base of the crypts, the higher the maturity rate of the intestinal epithelial cells, the enhanced secretory capacity, and the higher the absorptive capacity. Histomorphometric analyses showed superior villus architectural preservation in the NI groups (particularly NI800), suggesting enhanced enterocyte proliferation and mucosal repair. These findings were reinforced by upregulated expression of tight junction proteins (e.g., claudin, occludin), underscoring the role of NI in fortifying the intestinal barrier function. Similar improvements in intestinal morphology have been observed in studies using other probiotics and prebiotics (44). SCFAs are beneficial metabolites produced by anaerobic bacteria in the intestine when they degrade dietary fiber in food. They can provide a large amount of energy for animals, promote digestion, regulate the pH value of the intestine and other physiological functions (45, 46). Additionally, NI significantly altered the cecal SCFA profile, the NI600 group showed elevated acetate, propionate, and valerate concentrations. These microbial metabolites serve as energetic substrates for colonocytes and exhibit pleiotropic anti-inflammatory and immunoregulatory properties (47). The increase in SCFAs suggests that NI may promote the growth of beneficial bacteria in the gut. Research has shown that SCFAs can improve gut health and enhance immune function (48).

Tight junction proteins are a significant part of intestinal epithelial cells, and the intestinal mechanical barrier formed by these cells protects the body from exogenous pathogenic bacteria and entry of substances, such as toxins. The intestinal barrier also selectively allows some nutrients and water to enter the body (49). Disruption of the tight junction barrier impairs the function of the epithelial barrier, leading to the occurrence of some intestinal diseases (50). Tight junction proteins are typically utilized as indicators for assessing the integrity and permeability of the intestinal mucosa (51, 52). ZO-1, occludin, and claudin are the most important tight junction proteins, which are closely related to maintaining the function of the intestinal mucosal barrier. The results of this experiment show that adding NI600 to the diet of rabbits significantly increased the relative expression of claudin mRNA in the jejunum and ileum. Adding NI800 significantly increased the relative expression of claudin mRNA in the jejunum. Adding NI1000 significantly increased the relative expression of occludin mRNA and claudin mRNA in the jejunum. This indicates that adding NI at various doses can enhance the expression of tight junction proteins in the intestine of rabbits to different extents, as well as strengthen the function of the intestinal barrier.

Microbiome diversity metrics revealed that NI (especially NI800) significantly enriched the Firmicutes phylum, which is intrinsically linked to polysaccharide metabolism and SCFA production. Linear discriminant analysis effect size (LEfSe) further identified NI-induced enrichment of functionally specialized taxa (e.g., Lachnospira), suggesting targeted remodeling of the gut microbiome to enhance host metabolic health. This shift in the gut microbiota composition could explain some of the observed beneficial effects of NI. Research has demonstrated that Lachnospira species are associated with improved gut health and SCFA production (53).

In conclusion, dietary NI supplementation, particularly at 800 mg/kg, showed promise as a natural feed additive for improving the growth performance, lipid and protein metabolism, antioxidant status, intestinal health, and gut microbiota composition in rabbits. Further research is needed to delineate the precise mechanisms underlying these beneficial effects and to optimize the utilization of NI in animal feed. Regulation of the gut microbiota and enhancement of intestinal barrier function appear to be key factors contributing to the overall health benefits of NI supplementation (54).

## 5 Conclusion

Dietary feed with NI, particularly at doses of NI800 and NI1000, significantly enhanced growth performance in rabbits, as evidenced by the increased final body weight, ADG, and ADFI. NI was demonstrated to improve metabolic health by decreasing serum cholesterol and urea levels, boosting antioxidant enzyme activities (SOD and GSH-Px), and lowering lipid peroxidation (MDA). It also enhanced intestinal health through elevated digestive enzyme activities (α-amylase and trypsin), improved villus morphology, and upregulated expression of tight junction proteins (claudin and occludin). Furthermore, NI was found to modulate the cecal microbiota, enriching SCFA-producing taxa and functional pathways linked to energy and nutrient metabolism. Collectively, these results of this study demonstrate that NI is a viable alternative to antibiotics for promoting health and productivity in rabbit production systems.

## Data Availability

The raw 16S rRNA sequencing data have been deposited into the NCBI Sequence Read Archive (SRA) under the BioProject accession number PRJNA1359562.
