# Supplementary material for: Effects of dietary nisin supplementation on the growth performance, serum biochemistry, digestive enzyme activities, intestinal morphology, and intestinal microbiota in rabbits
Source: Front Vet Sci. 2025 Dec 11;12:1726365. doi: 10.3389/fvets.2025.1726365 (PMC12739866; doi:10.3389/fvets.2025.1726365)
Supplement: Supplementary file 1 [file Table_1.docx]

1. **Supplementary Material**

Supplementary Table 8: Histopathological scoring criteria for rabbit small intestine (H&E staining)

| Parameter | Score 0 (Normal) | Score 1 (Mild) | Score 2 (Moderate) | Score 3 (Severe) |
| --- | --- | --- | --- | --- |
| Inflammatory Cell Infiltration | Normal, scattered inflammatory cells in lamina propria | Mild, focal increase in inflammatory cells within the lamina propria | Moderate, diffuse infiltration extending into the submucosa | Severe, transmural infiltration with numerous neutrophils and/or microabscesses |
| Epithelial Damage | Intact villous and crypt architecture | Mild epithelial degeneration/erosion at the villous tip | Villous blunting and fusion, noticeable epithelial sloughing | Frank ulceration, complete loss of mucosal epithelium |
| Crypt Alterations | Normal crypt structure | Mild crypt hyperplasia/elongation | Crypt distortion, branching, or mild atrophy | Severe crypt loss or destruction |
| Edema & Congestion | No edema or vascular dilation | Mild edema and/or vascular congestion in the submucosa | Moderate edema and vascular congestion | Severe edema with marked tissue separation |
| Goblet Cell Population | Normal density | Mild reduction | Marked reduction | Nearly complete absence |

The total histopathological score is the sum of all individual parameter scores (Maximum possible score = 15).
